# Ocular Imaging Challenges, Current State, and a Path to Interoperability: A HIMSS-SIIM Enterprise Imaging Community Whitepaper

**DOI:** 10.1007/s10278-024-01261-0

**Published:** 2024-10-01

**Authors:** Kerry E. Goetz, Michael V. Boland, Zhongdi Chu, Amberlynn A. Reed, Shawn D. Clark, Alexander J. Towbin, Boonkit Purt, Kevin O’Donnell, Marilyn M. Bui, Monief Eid, Christopher J. Roth, Damien M. Luviano, Les R. Folio

**Affiliations:** 1 Office of Data Science and Health Informatics, National Eye Institute, National Institute of Health, Bethesda, MD USA; 2 Massachusetts Eye and Ear, Harvard Medical School, Boston, MA USA; 3 Verana Health, Inc, San Francisco, CA USA; 4 Cybersecurity-Network Protection, Cleveland Clinic Foundation, Cleveland, OH USA; 5 Department of Radiology, Cincinnati Children’s Hospital, University of Cincinnati College of Medicine, Cincinnati, OH USA; 6 Uniformed Services University of the Health Sciences, Bethesda, MD USA; 7 Canon Medical Research, Mayfield, KY USA; 8 Departments of Pathology and Machine Learning, H. Lee Moffitt Cancer Center & Research Institute, Tampa, FL 33612 USA; 9 Ministry of Health, Riyadh, Saudi Arabia; 10 Duke Health, Durham, NC USA; 11 Virginia Tech Carilion School of Medicine, University of Virginia School of Medicine, Roanoke, VA USA; 12 Diagnostic Imaging & Interventional Radiology, Department of Machine Learning, H. Lee Moffitt Cancer Center & Research Institute, Tampa, FL USA

**Keywords:** Ophthalmology, Optometry, DICOM, Interoperability, Enterprise imaging, Medical imaging, Informatics

## Abstract

Office-based testing, enhanced by advances in imaging technology, is routinely used in eye care to non-invasively assess ocular structure and function. This type of imaging coupled with autonomous artificial intelligence holds immense opportunity to diagnose eye diseases quickly. Despite the wide availability and use of ocular imaging, there are several factors that hinder optimization of clinical practice and patient care. While some large institutions have developed end-to-end digital workflows that utilize electronic health records, enterprise imaging archives, and dedicated diagnostic viewers, this experience has not yet made its way to smaller and independent eye clinics. Fractured interoperability practices impact patient care in all healthcare domains, including eye care where there is a scarcity of care centers, making collaboration essential among providers, specialists, and primary care who might be treating systemic conditions with profound impact on vision. The purpose of this white paper is to describe the current state of ocular imaging by focusing on the challenges related to interoperability, reporting, and clinical workflow.

## Introduction

Diagnostic testing of the eye spans multiple modalities in multiple practice settings. While many of the testing modalities are mature, the end-to-end clinical workflows are needed to consistently “capture, index, manage, store, distribute, view, exchange, and analyze images” [1]. Using a clinical use case, our aim is to provide an overview of ocular imaging and highlight many of the current challenges. After describing these challenges, we will discuss current efforts that provide a way forward to standardize image capture, storage, and distribution.

## Current State of Eye Care Practice

### Framing Use Case

A 55-year-old man was followed for over 20 years by several eye clinics for routine eye care and for retinal photography for a choroidal nevus in his left eye. At one point, he developed rapidly deteriorating vision in his left eye and sought care. He complained to his optometrist that his distance vision only improved when he used the lower segment of his bifocals; in other words, he required a more hyperopic prescription. Two weeks later, he again experienced decreased vision in his left eye, so he returned to his eye care provider who again changed his prescription. A few weeks later, he was still noticing deterioration in the vision of his left eye and his optometrist increased the prescription power of the eyeglasses a third time.

**Fig. 1:**
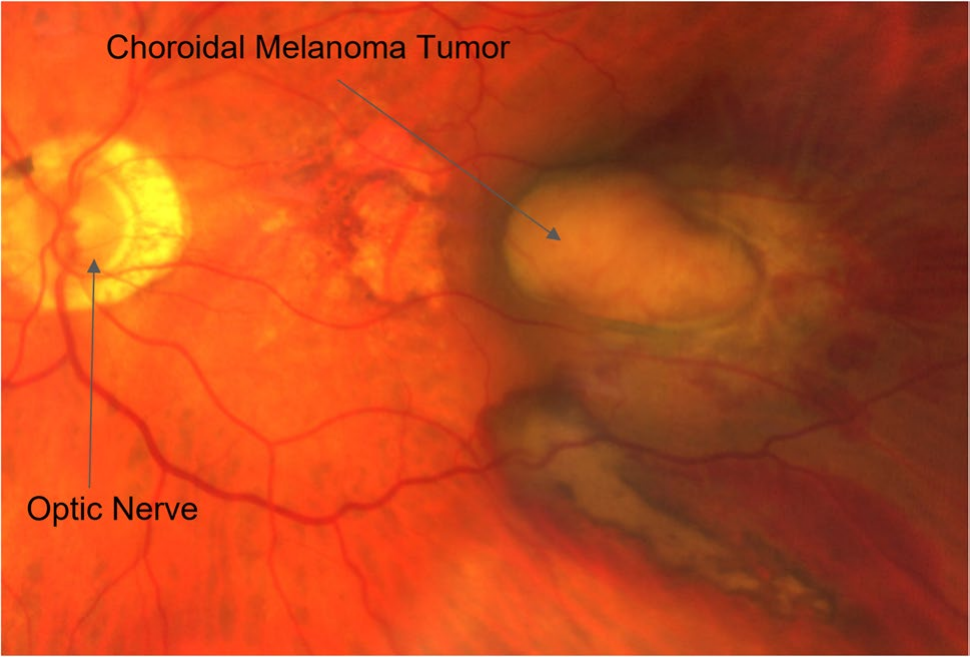
Ophthalmic fundus photograph of the left eye: leftmost arrow: optic nerve, rightmost arrow: pigmented lesion (choroidal melanoma) projecting into the vitreous

After three new eyeglass prescriptions in less than 2 months, and with his vision continuing to deteriorate, the patient sought a second opinion. At the ophthalmology clinic, he underwent dilation of his pupils and color retinal fundus photography, which revealed a nevus concerning for choroidal melanoma (Fig. 1). Because the risk of transformation from a benign nevus to malignant melanoma is about 1 in 5000, the ophthalmologist requested prior color retinal photographs to confirm suspected changes in the nevus. The prior eye care providers were not able to provide the images for a variety of reasons, including misunderstanding of privacy issues, poor quality of images, inability to send images due to technical reasons, and outright loss of the original images. While it is common for images to be shared between practices via low-resolution, black-and-white faxes or printed reports, in this case, none of the prior eye care providers had an imaging archive, and none had provided the patient with images or reports in paper or electronic format. The use of printouts in healthcare is generally problematic but for image sharing is especially problematic due to the need for accurate color representation which is critical to this case.

**Fig. 2:**
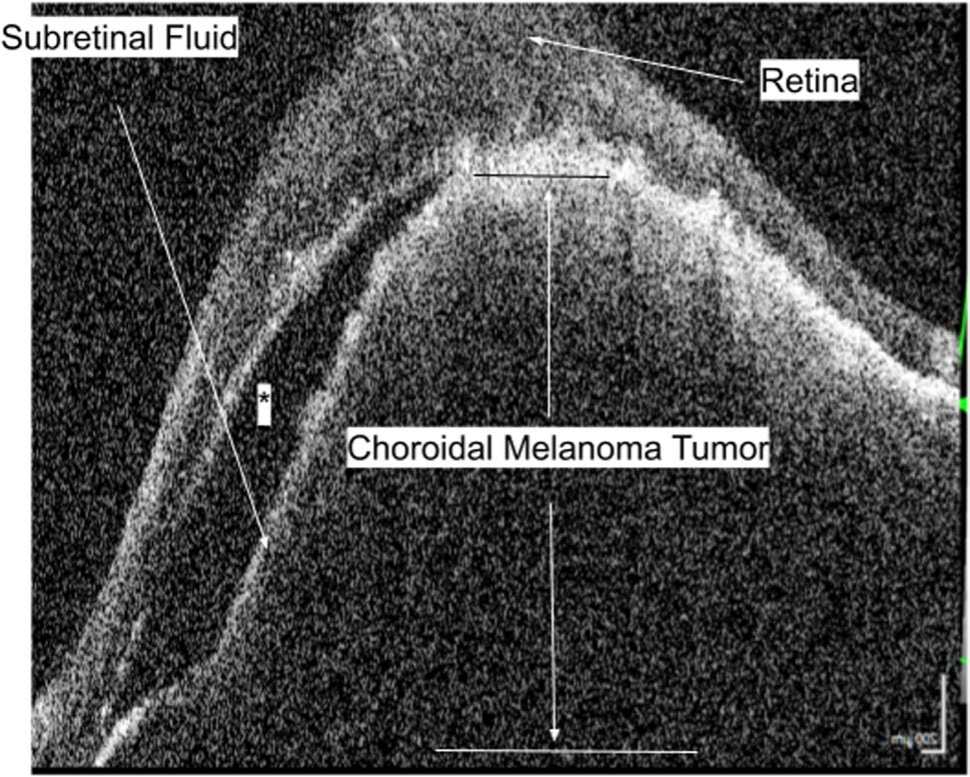
Left eye, optical coherence tomography. The cross-section of the lesion reveals that the growth of the choroidal melanoma tumor is causing extreme elevation of the retina causing a serous retinal detachment. The change in eye anatomy changed the optics of the eye and caused rapidly changing prescription

**Fig. 3:**
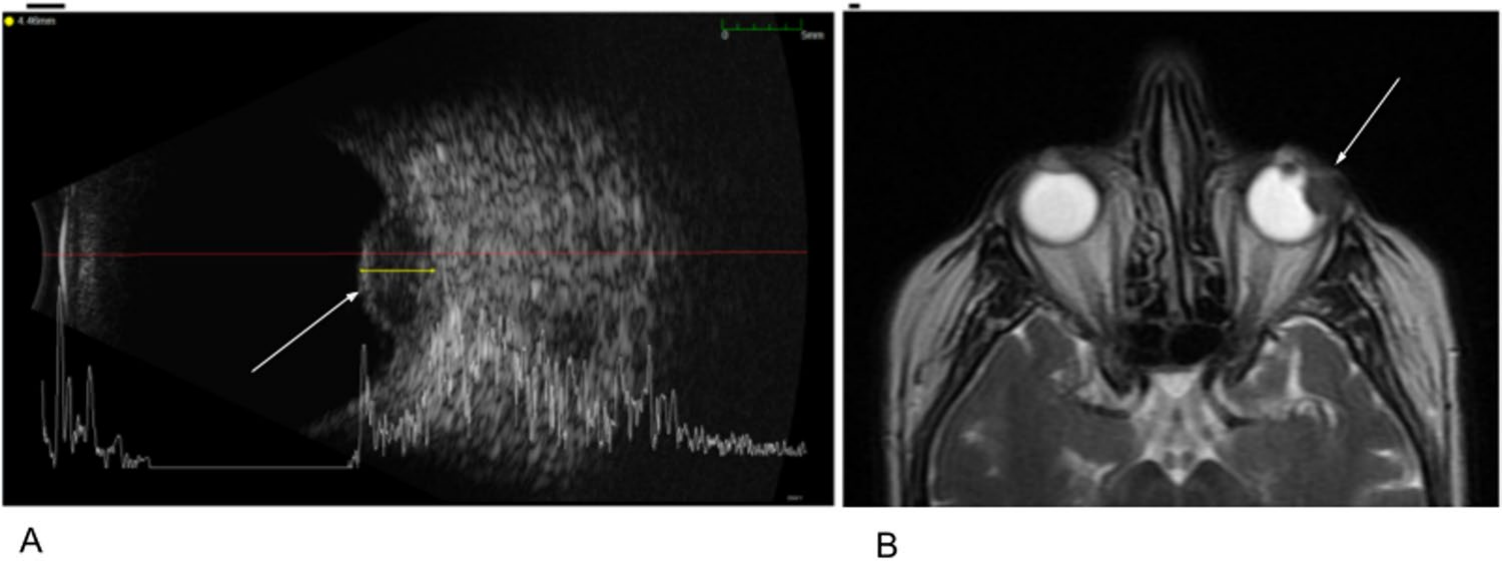
**A** Left eye, ophthalmic ultrasound of choroidal melanoma (arrow). Note the elevated nature of the lesion in the retina projecting into the vitreous, the gel-like fluid that fills the eye. **B** Left eye, T2-weighted MRI of choroidal melanoma (arrow). Note the low signal intensity lesion and elevation

Further imaging at the ophthalmology clinic with ultrasound and optical coherence tomography (OCT) revealed an elevated lesion under the retina and a serous retinal detachment involving the macula (Fig. 2). Ophthalmic ultrasound and MRI (Fig. 3) of the left eye showed a raised hypoechoic lesion on ultrasound with low T2 signal intensity on MRI supporting a diagnosis of choroidal melanoma. In this case, the retinal detachment changed the optics of the eye, rapidly changing the eyeglasses prescription. Fortunately for this patient, the tumor did not metastasize, and with radiation therapy, the tumor regressed completely.

### Lessons from the Case

This case exemplifies how challenging it can be for providers to understand the clinical presentation of a patient by simply looking at one image at one point in time. If eye care providers could look at the progression of the pigmented lesion over time, patients like this might receive an earlier diagnosis, which can be vision or lifesaving. The improved adoption of standards and use of imaging informatics tools will enable eye care providers to have more comprehensive information, leading to earlier diagnoses. This need for comparison studies is especially true for diseases requiring long-term monitoring, such as glaucoma and macular degeneration. Additionally, when ocular imaging data is stored and managed in a standard manner, new tools, such as artificial intelligence (AI) and clinical alerts, can be further enhanced.

### Ocular Tests and DICOM Representation

There are five main goals of ocular testing: screening, diagnosing, progression monitoring, procedure planning, and clinical documentation. Reliance on in-office testing is critical for all common blinding eye diseases including cataract, glaucoma, diabetic retinopathy, and age-related macular degeneration. The testing modalities can be categorized by purpose and anatomic region (Table 1). The Digital Imaging and Communications in Medicine (DICOM) Working Group 9 (WG-9) focuses on eye care and has developed standards for many of the modalities commonly used in the eye clinic (see Table 2).

**Table 1:** Broad areas of ocular testing including modalities and their anatomic region of focus

Category	Purpose	Modality or clinical procedure	Anatomic region
Photography	Structure	Camera	External eye, anatomy, anterior segment, posterior chamber
Computerized imaging	Structure	Optical coherence tomography (OCT)	Anterior segment, posterior segment
	Structure	Optical coherence tomography angiography (OCT-A)	Posterior segment
	Structure	Confocal scanning laser ophthalmoscopy	Anterior segment, posterior segment
Angiography	Structure	Fluorescein (FA)	Posterior segment
	Structure	Indocyanine green (ICG)	Posterior segment
Ultrasound	Structure	A-scan (ocular measurements)	External eye, anatomy, anterior segment, posterior chamber
	Structure	B-scan	External eye, anatomy, anterior segment, posterior chamber
Optical biometry	Structure	Measurements in anticipation of cataract surgery	Anterior segment
Topography	Structure	Scanning slit lamp	Cornea
Visual acuity	Function	Snellen, Random E, ETDRS, etc	Entire visual system
Contrast sensitivity	Function	Pelli-Robson	Entire visual system
Perimetry (visual field)	Function	Goldmann, Humphrey, Octopus	Entire visual system

**Table 2:** Current testing modalities and data reports relevant to eye care and defined by the DICOM standard

Imaging modality
Ophthalmic photography
Optical coherence tomography (OCT)
Optical coherence tomography angiography (OCTA)
Wide field ophthalmic photography
Ultrasound
Visible light
Digital X-ray
CT/Enhanced CT (with contrast)
MR/enhanced MR (with contrast)
Confocal microscopy
Data reporting
Macular grid thickness structured report
Ophthalmic refractive measurements
Spectacle prescription report
Ophthalmic axial measurements
Ophthalmic visual field
Ophthalmic thickness map
Corneal topography map
Ophthalmic OCT B-scan volume analysis
Ophthalmic OCT en face
Heightmap segmentation (OCT retinal layers)

In the eye care setting, some imaging workflows are visit-based, with the care provider deciding in the visit what testing is needed; however, most imaging workflows are order-based. An order for an ocular test commonly results in several data objects. There is an image object which is viewable in an image viewer. Today, the viewer is often proprietary software that is, in some cases, linked to a specific vendor-offered device. Within an image viewer, the user may be able to toggle between image series objects, look across images captured at different time points, and do in-software measurements of key features of the structure being imaged (ex., retinal nerve fiber layer on OCT or cup-to-disc ratio on color fundus image). In its native format, the image includes metadata attributes for elements such as ordering details (dates, ordering clinician, laterality, device, and modality), patient details (name and record number), pixel-level details (columns, rows, and color values), and other details that are captured before and during testing. The test output often includes a structured report, usually in portable document format (pdf), that highlights the key elements used to drive decision-making for the patient. Like the images, the report content and key measures differ by device manufacturer. At this time, the data in structured reports lack standardization across vendors.

### Clinical Imaging Workflow

**Fig. 4:**
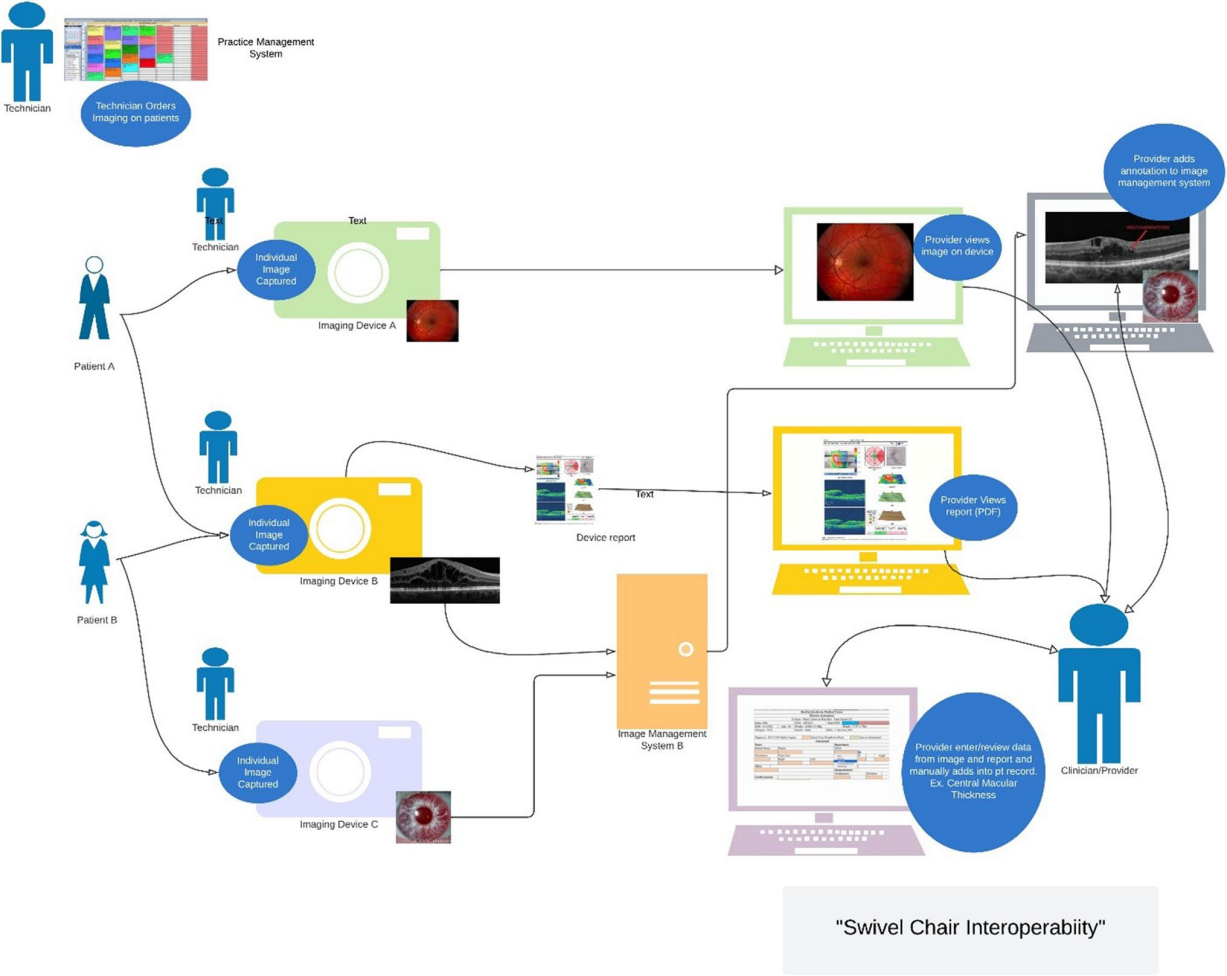
Example of an ocular imaging environment including a practice management system (PMS) (for scheduling and patient demographics); multiple imaging modalities unconnected to the PMS, often with its own image storage, image display, and clinical documentation components; and an eye care provider required to interact with several modality clinical documentation components in what has been coined “swivel chair interoperability”

The clinical imaging workflow in routine eye care is fragmented and siloed. Providers must interact with several workstations, clinical applications, and interfaces while simultaneously caring for patients. The diagram in Fig. 4 is a generalized depiction of the digital systems, interfaces, data flow, and communication/messaging in an eye care setting. In our example, the computer that the provider uses for ordering imaging is not integrated with the workstation that is utilized to manage and document patient care, including the results of in-office testing, treatment, or ocular surgery. In other words, the imaging modalities, image management systems, and electronic medical records lack interoperability. The provider may need to have multiple applications open during a visit to manually enter data from the image, annotation, or structured clinical report into an electronic medical record (EMR), with the inherent error rate of manual transcription. This workflow has been referred to as “swivel chair interoperability” (user is responsible for copying and translating data while swiveling from one screen/system to another). Further, a query for a specific patient is generally accomplished by searching the EMR for details needed to separately query an image management system for individual file retrieval. The lack of full system integration can lead to incomplete review of all imaging modalities resulting in quality of care and patient safety issues and significant barriers to efficient collaborative care and research. Even if these issues do not occur in a majority of practices, with the fact that clinicians perform more than 3 million cataract surgeries, 7 million intravitreal injections, and tens of millions of in-office tests per year, the impact of interoperability issues is large.

**Fig. 5:**
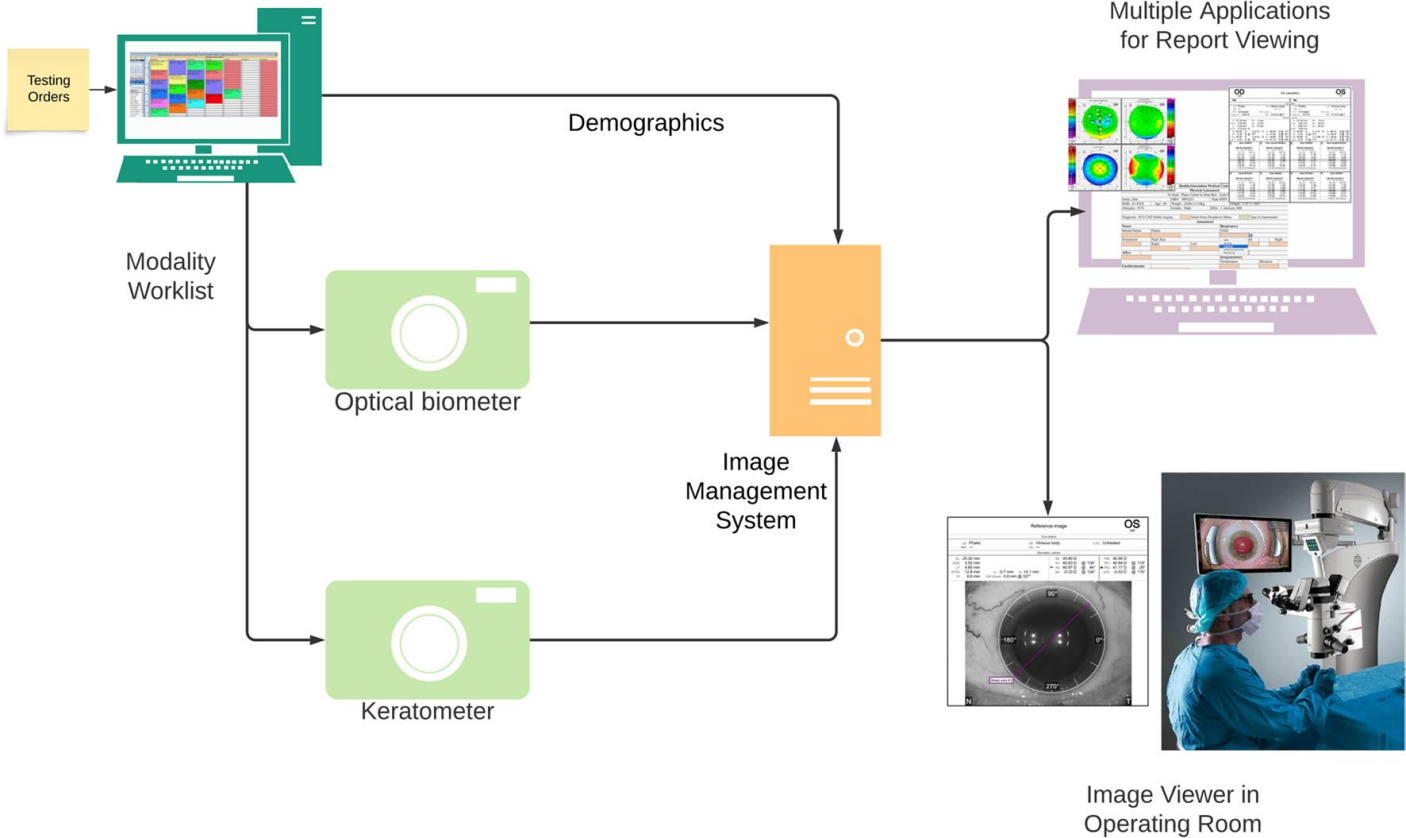
Example of the current state of an ocular imaging workflow for a patient being evaluated for cataracts in an operating room

Much of this inefficiency could be solved by using a DICOM-based approach as shown in Fig. 5. In this ideal state, the provider orders imaging followed by the technologist/photographer selecting the patient on the device using a DICOM modality worklist, which facilitates accurate patient identification and demographics. After performing imaging, the raw images, data, and summary report are sent to an imaging archive and a link is added to the EMR. The provider can then review the imaging studies using either an enterprise or eye care–specific picture archiving and communication system or diagnostic viewer [2]. Studies can be forwarded to consulting or tertiary care entities using standard DICOM and HL7/Fast Healthcare Interoperability Resources (FHIR) messaging capabilities. Today, portions of this workflow exist at some organizations. However, many eye care modalities are unable to accept a DICOM modality worklist and do not release the acquired images without added licenses, instead sending only the summary report in PDF form, which is not in a machine-readable format.

The fact that eye care image and data repositories exist outside of both enterprise EMR systems and enterprise imaging archives is another problem in the eye care community. Having siloed eye care solutions prevents providers from holistically reviewing patient data acquired by other specialties, and the siloed solutions may lack the robust backups, security, and data exchange capabilities that are commonly provided by enterprise systems. This can lead to inefficiency and, at times, impact the ability to provide high-quality care. The lack of EMR and imaging data archive integration is due to the large percentage of independent providers that dominate the specialty. These practices often do not feel the need to obtain expensive image and data management systems and to engage with the rest of the healthcare system. This practice landscape has encouraged device manufacturers to create proprietary image databases rather than routinely deploying a standard-based approach to storage and distribution. This non-standard approach has a negative downstream impact. For example, because imaging studies are stored on a single modality, there is no need to apply study descriptors to the study. The lack of descriptors contributes to interoperability challenges and compromises patient care and safety [1].

Moving to the DICOM standard and protocols has been lagging in eye care despite the availability of standards for the most common test modalities. Vendors have been slow to produce DICOM solutions due to concerns about making ocular imaging data and analysis widely available which can be perceived as a loss of control, a dearth of DICOM expertise, and the lack of a clear business case for executives to commit to devoting the significant development effort required. The flexibility and scope of the DICOM standard have allowed vendors to claim DICOM conformance even if they are simply generating encapsulated PDF documents of their proprietary analyses (and no corresponding standard-based data). The lack of full-fidelity imaging data and the reliance on encapsulated PDFs will hamper future innovation and AI development in the eye field and beyond.

### Pathway Toward Interoperability

#### DICOM in Eye Care

Efforts toward standardization in ophthalmology are following the example set by the field of radiology. For example, the DICOM Working Group 9 (WG-9) started work on ophthalmology standards in 1998 [3] and subsequently developed standards for the most common ophthalmic testing modalities. Despite more than two decades of effort, further action toward fully adopting DICOM and interoperability standards in eye care is still needed. In 2022, WG-9 reconvened after a 3-year hiatus to provide a forum in which to prioritize new standards, identify gaps in vendors’ implementation of standards, and update the current DICOM standard to ensure it reflects current technology and reporting [4]. As an initial effort to improve adoption, the American Academy of Ophthalmology has compiled and posted a list of device DICOM conformance statements on their website [5].

Presently, adherence to the DICOM standard is not required for market authorization of ophthalmic imaging devices by the FDA, and even those currently marketed as “DICOM-compliant” may not provide the raw data needed for the most impactful applications (as mentioned above). However, in May 2022, the FDA added DICOM to the recognized consensus standards for the ophthalmic specialty task group. This allows device manufacturers to declare conformance to meet regulatory requirements and hopefully will encourage manufacturers to use DICOM conformance to accelerate the time to market [6].

#### Interoperability Work Being Done Outside of DICOM

While DICOM is a useful standard in medical imaging, it is not sufficient to address all interoperability challenges in data collection and management. Interoperability standards, such as HL7 Fast Healthcare Interoperability Resources (FHIR), are one effort to facilitate data interoperability between health care systems. Adoption of technologies to enable connected systems of exchange is equally essential to ensuring the best care. Adoption of ocular testing standards presents an opportunity to address many key barriers to promote collaboration. FHIR accomplishes this by directly exposing discrete data elements as services. The ImagingStudy FHIR resource, developed jointly by HL7 and DICOM, provides an EHR index entry for a patient procedure with links to access pre-rendered JPEG images for easy display via DICOMweb [7, 8]. In 2021, there was initial development of an implementation guide for FHIR for ophthalmology and a corresponding Connectathon in which several device manufacturers participated; however, more work is needed to advance this work toward maturity. [9, 10]

Another approach to interoperability relies on Integrating the Healthcare Enterprise (IHE). The IHE Eye Care [11] domain has published several integration profiles that describe how to implement standards for eyecare-specific workflows. The IHE Eye Care domain was formed in 2005, and their mission is to bring together information technology stakeholders and healthcare professionals to implement standards for communicating patient information efficiently throughout healthcare facilities. Sustained support of DICOM WG-9 and the IHE Eye Care Domain is paramount. In 2021, a HIMSS-SIIM white paper included ocular imaging as one of the key enterprise imaging modalities benefitting from standardization and interoperability in the context of interactive multimedia reporting [12]. The same community published another white paper in 2022 about the technical challenges that led to an IHE profile. [13, 14]

#### US Federal Government Efforts

To assess the need for interoperability in eye care and eye-related research, the National Eye Institute (NEI), part of the National Institutes of Health (NIH), issued a Request for Information regarding ocular imaging standards in February 2022 [15]. A few months later, the NEI, the US Food and Drug Administration (FDA), and the Office of the National Coordinator for Health Information Technology (ONC) jointly supported a workshop to develop and implement a plan to improve vision research and clinical care through ocular imaging standard adoption. This workshop brought together researchers; clinicians; policymakers; and representatives of vendors, government, research sponsors, and professional societies to collaboratively identify barriers and approaches toward widespread adoption of standards for interoperability in ophthalmology [16].

NEI, FDA, and ONC committed to collaboratively using their leverage to effect real-world interoperability in the eye care community. This workshop represented an important step toward that goal and has been followed by other important policy shifts. For example, the US Department of Defense and Department of Veterans Affairs, two of the largest health systems in the USA, have begun to incorporate interoperability requirements into their purchasing plans. Additionally, the NEI recognizes DICOM as a recommended standard for ocular image data sharing in Data Management and Sharing (DMS) Plans, now required for funded projects according to the NIH DMS Policy. [17–19]

ONC issued the United States Core Data for Interoperability (USCDI) to classify current capabilities regarding many aspects of the healthcare system and provide a standard mandate for core data elements that must be able to be exchanged in certified health systems. At present, ocular data consisting of intraocular pressure, visual acuity, and refraction would be included in the fields related to clinical exams that must be exchanged. Version 3 of the USCDI includes diagnostic imaging test and diagnostic imaging report [20]. Key measurements obtained in ocular imaging, such as cup-to-disc ratio for glaucoma and central macular thickness for age-related macular degeneration, are often measured within the vendor analysis software but stored in proprietary DICOM elements that never make it to the electronic health record. By including key imaging measures in the USCDI, technology developers would be incentivized to ensure that these important data elements can be incorporated within EHRs and exchanged across sites.

In an effort to place the patient in the center of health care, the 21st Century Cures Act included rules to prevent information blocking [21]. This act called for putting the patient first in health technology and required healthcare systems to provide transparency into the cost and outcomes of their care and enabled the creation of smartphone apps to provide patients convenient access to their records and an app economy to enable innovation and choice. The inability to exchange patients’ ocular imaging data should be considered a form of information blocking, since they are key indicators of health.

## Conclusion

Eye care must fully adopt a standard-based approach in ocular diagnostics to optimize the clinical and research applications to include imaging data following DICOM and FDA consensus standards. To reach that goal, eye care professionals must implement tools for non-proprietary image storage and sharing at both the enterprise and private practice scale. To achieve interoperability, all participants in the care delivery process must be engaged, from vendors to practitioners. This participation can be accomplished with vendor-neutral archives and imaging archives with the ability to share among trusted partners via standardized messaging and tools, including HL7/SMART on FHIR. We encourage and challenge eye care providers, device manufacturers, and technology developers to engage in collaboration with professional societies including Connectathons and hackathons.

The eye care and research communities are long overdue for the adoption of DICOM-based imaging. Full engagement is the rate-limiting step in achieving a future of interoperability that would improve patient safety, care quality, and access to health records. However, the specialty cannot move forward without the collaboration of manufacturers, ophthalmologists, and the informatics community.
